# P-470. CD4 Testing in HIV Care: Uncovering Patterns of Over-Monitoring

**DOI:** 10.1093/ofid/ofae631.669

**Published:** 2025-01-29

**Authors:** Nouf K Almaghlouth, Mallikarjun Komsani, Anuoluwapo Shobayo, Diane Ayuninjam, Elena Morel, Joseph M Garland, Fizza S Gillani, Timothy P Flanigan

**Affiliations:** Warren Alpert Medical School of Brown University, Providence, Rhode Island; Warren Alpert Medical School of Brown University, Providence, Rhode Island; Warren Alpert Medical School of Brown University, Providence, Rhode Island; Warren Alpert Medical School of Brown University, Providence, Rhode Island; Warren Alpert Medical School of Brown University, Providence, Rhode Island; Immunology Center, The Miriam Hospital, RI; Brown Alpert Medical School, Providence RI, USA, Providence, Rhode Island; The Miriam Hospital and Brown University, Providence, Rhode Island

## Abstract

**Background:**

In the realm of HIV care, CD4 monitoring guidelines serve as a cornerstone for managing the health and treatment plans of individuals living with HIV. Various authoritative bodies, including the U.S. Department of Health and Human Services (HHS) offer nuanced guidelines tailored to the latest research and clinical best practices. In our study, we aimed to investigate the frequency of unnecessary CD4 testing and assess healthcare providers' comfort level of less frequent monitoring.

**Figure 1:**
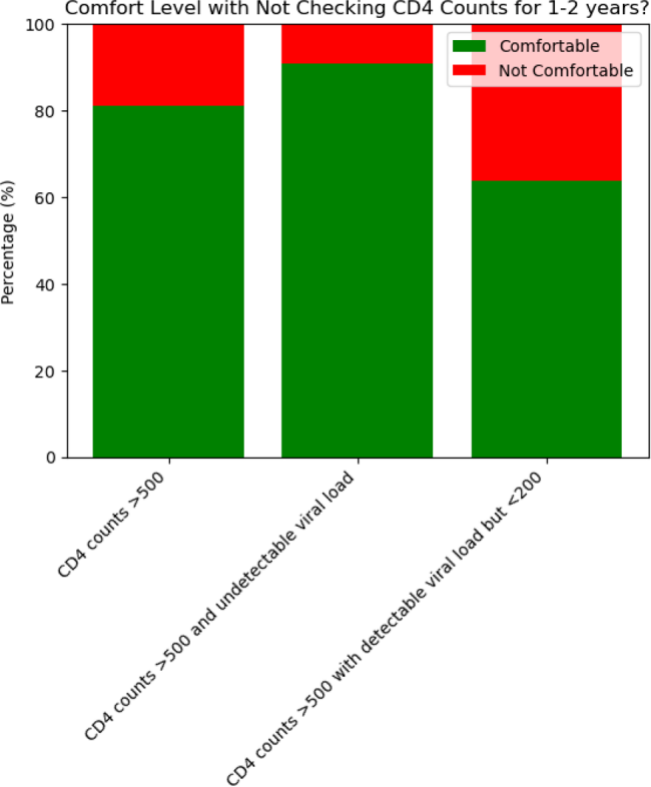
Healthcare Provider Comfort Levels with Deferring CD4 Count Testing above 500

**Methods:**

We conducted a retrospective cohort study of HIV patients under active care at the Lifespan Immunology Clinic, Miriam Hospital, Providence, Rhode Island, from 2021 to 2023, with undetectable viral loads (< 200 VL) and CD4 counts over 500. Data were extracted from electronic health records, including demographics and clinical measures. Additionally, we conducted qualitative surveys among key healthcare providers to determine adherence to guidelines and to identify any patterns of unnecessary CD4 testing.

**Figure 2:**
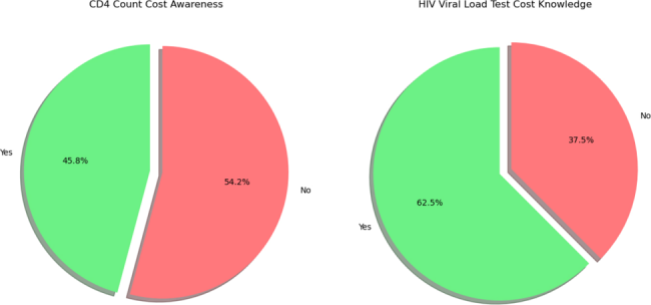
Awareness of Costs Associated with CD4 Count and HIV Viral Load Testing Among Healthcare Providers

**Results:**

In our first phase of the study, a cohort of 1,929 active unique patients was monitored for their immunological status in 2021. Among these, 1,226 individuals underwent CD4 testing as the event date, with 825 achieving CD4 counts >500. Further analysis highlighted a subgroup of 712 patients who exhibited undetectable viral loads over a three-year span with CD4 counts >500 in 2021. Additionally, 610 of these patients continued to undergo regular CD4 testing after the event date. This group represents approximately 86% of those who were undetectable from 2021 to 2023. In the second phase of the study, a survey involving 40 healthcare providers at the immunology clinic assessed practices in monitoring with CD4 counts >500. The survey revealed 77% were comfortable not performing further CD4 tests, regardless of the patient's viral load status and 90% preferred not testing patients with undetectable viral loads for 1-2 years.

**Conclusion:**

Our study observed significant increasing frequency with unnecessary monitoring of CD4 above 500 with undetectable viral loads. This highlights the importance of implementing future guidelines for minimizing CD4 testing in stable patients, aligning clinical practices and improving outcomes.

**Disclosures:**

**All Authors**: No reported disclosures

